# Effects of ocean acidification on primary production in a coastal North Sea phytoplankton community

**DOI:** 10.1371/journal.pone.0172594

**Published:** 2017-03-08

**Authors:** Tim Eberlein, Sylke Wohlrab, Björn Rost, Uwe John, Lennart T. Bach, Ulf Riebesell, Dedmer B. Van de Waal

**Affiliations:** 1 Alfred Wegener Institute, Helmholtz Centre for Polar and Marine Research, Am Handelshafen 12, Bremerhaven, Germany; 2 Helmholtz Institute for Functional Marine Biodiversity Oldenburg (HIFMB), Carl von Ossietzky Straße, Oldenburg Germany; 3 GEOMAR Helmholtz Centre for Ocean Research Kiel, Düsternbrooker Weg 20, Kiel, Germany; 4 Netherlands Institute of Ecology (NIOO-KNAW), Wageningen, The Netherlands; Auckland University of Technology, NEW ZEALAND

## Abstract

We studied the effect of ocean acidification (OA) on a coastal North Sea plankton community in a long-term mesocosm CO_2_-enrichment experiment (BIOACID II long-term mesocosm study). From March to July 2013, 10 mesocosms of 19 m length with a volume of 47.5 to 55.9 m^3^ were deployed in the Gullmar Fjord, Sweden. CO_2_ concentrations were enriched in five mesocosms to reach average CO_2_ partial pressures (*p*CO_2_) of 760 μatm. The remaining five mesocosms were used as control at ambient *p*CO_2_ of 380 μatm. Our paper is part of a PLOS collection on this long-term mesocosm experiment. Here, we here tested the effect of OA on total primary production (PP_T_) by performing ^14^C-based bottle incubations for 24 h. Furthermore, photoacclimation was assessed by conducting ^14^C-based photosynthesis-irradiance response (P/I) curves. Changes in chlorophyll *a* concentrations over time were reflected in the development of PP_T_, and showed higher phytoplankton biomass build-up under OA. We observed two subsequent phytoplankton blooms in all mesocosms, with peaks in PP_T_ around day 33 and day 56. OA had no significant effect on PP_T_, except for a marginal increase during the second phytoplankton bloom when inorganic nutrients were already depleted. Maximum light use efficiencies and light saturation indices calculated from the P/I curves changed simultaneously in all mesocosms, and suggest that OA did not alter phytoplankton photoacclimation. Despite large variability in time-integrated productivity estimates among replicates, our overall results indicate that coastal phytoplankton communities can be affected by OA at certain times of the seasonal succession with potential consequences for ecosystem functioning.

## Introduction

Atmospheric CO_2_ partial pressure (*p*CO_2_) is currently rising at an unprecedented rate due to anthropogenic activities. This leads to enhanced CO_2_ uptake by the oceans and a decrease in ocean surface water pH, referred to as ocean acidification (OA) [1,2]. From 1765 until 1994, pH values were calculated to have already decreased by 0.08 units. Present-day CO_2_ concentrations of around 400 μatm are predicted to more than double by the year 2100, which will result in a further acidification of the ocean [3]. After the Polar Oceans, the North Atlantic is expected to show strongest changes in response to rising *p*CO_2_ [3,4]. As a major sink of anthropogenic CO_2_, the North Atlantic Ocean basin stores almost a quarter of the global oceanic anthropogenic CO_2_, although covering only 15% of the global ocean area [5]. The projected changes in ocean carbonate chemistry may thus not only have strong effects on the marine biota, but also on the oceanic carbon cycling.

Phytoplankton take up inorganic carbon (C_i_) in the photic zone and fix it into organic compounds, thereby providing a carbon and energy source for higher trophic levels. The key enzyme of carbon fixation, the CO_2_-binding enzyme Ribulose 1,5-bisphosphate Carboxylase/Oxygenase (RubisCO), exhibits a generally low affinity for its substrate CO_2_ [6–8]. To avoid C_i_ limitation, many phytoplankton species operate carbon concentrating mechanisms (CCMs) [9,10]. The efficiency in CO_2_ fixation depends on both the type of RubisCO as well as the mode of CCMs so that the response of phytoplankton to OA cannot be generalized across taxa [11–13]. Various studies have provided mechanistic insights into the CO_2_-dependent regulation of CCMs and thus CO_2_ fixation over a range of phytoplankton species (e.g. [14–16]). Besides species-specific differences, also strains of the same species may respond differently (e.g. [17–19]), which further complicates predictions on OA-driven changes in primary production.

To test these effects directly, numerous studies have exposed natural phytoplankton communities to high *p*CO_2_, either in bottle incubations or mesocosms, often finding higher rates of CO_2_ fixation under OA [20]. In these experiments, which lasted only a couple of days up to a month, the effects were yet relatively small. Here, we investigated the impact of OA on primary production by a natural phytoplankton community over an entire winter-to-summer succession. Experiments were performed in large scale mesocosms, deployed in the Gullmar Fjord located in Southwest Sweden at the Skagerrak coast in 2013 [21]. Depending on the wind direction and tides, the fjord consists of high saline bottom water from the North Atlantic, a low salinity thin surface layer fed with water from the river Örekil, and in between a layer fed by the Baltic current. Monitoring data from over 100 years have shown that the phytoplankton spring community in the Gullmar Fjord is typically dominated by diatoms, whereas summer blooms often comprise dinoflagellates [22,23]. We assessed primary production of the phytoplankton community from the mesocosms as well as the fjord by applying ^14^C incubations over 24 h [24]. We furthermore assessed the light dependency of CO_2_ fixation by performing photosynthesis-irradiance response curves in short incubations (80 min.).

## Material and methods

### Primary production experiments did not involve endangered or protected species

The KOSMOS 2013 mesocosm experiment was performed in the Gullmar Fjord (Kristineberg, Sweden) from March until July 2013 as part of the project BIOACID (Biological Impacts of Ocean ACIDification) phase II. Ten mesocosms were deployed near Kristineberg, with permission from the Sven Lovén Centre for Marine Infrastructure. The mesocosms were cylindrical polyurethane bags with a 2 m diameter mounted in a floatation frame [25]. The bags reached a depth of 17 m and were closed at the bottom with a 2 m long conical sediment trap [26]. Two days prior to the experiment (i.e. t-2), a water body was enclosed inside the mesocosms by lifting the upper end about one meter above the surface.

All mesocosms had a salinity of about 29, and nitrate, phosphate and silicate concentrations of about 7, 0.8, and 10 μmol L^-1^, respectively. CO_2_ enrichment was conducted on t-1 and t0, for which sterile-filtered and CO_2_-saturated seawater from the Gullmar Fjord was added to five mesocosms (M2, M4, M6, M7, M8). The remaining five mesocosms (M1, M3, M5, M9, M10) were treated as controls and received no CO_2_-enriched seawater. Average *p*CO_2_ (based on dissolved inorganic carbon (DIC) and spectrophotometric pH_T_ measurements) in the ‘low’ and ‘high’ CO_2_ treatments were about 380 and 760 μatm, respectively. The systems were open and allowed a gas exchange at the sea surface. To account for CO_2_ losses to the atmosphere by outgassing and for CO_2_ consumption by primary production, CO_2_ was added on a regular basis to the ‘high’ CO_2_ treatments. As a consequence, CO_2_ concentrations remained above the control treatment at all times (for more details see [21]). Sampling of seawater from each mesocosm was done with a depth-integrated water sampler (Hydro-Bios). After initial sampling on t0 and t1, samples were taken every other day until t109 (i.e. t3, t5, t7 etc.). For further information on the design and set-up of the experiment, as well as the CO_2_ perturbation and sampling techniques, we refer to [21].

### Sampling for primary production

**Fig 1:**
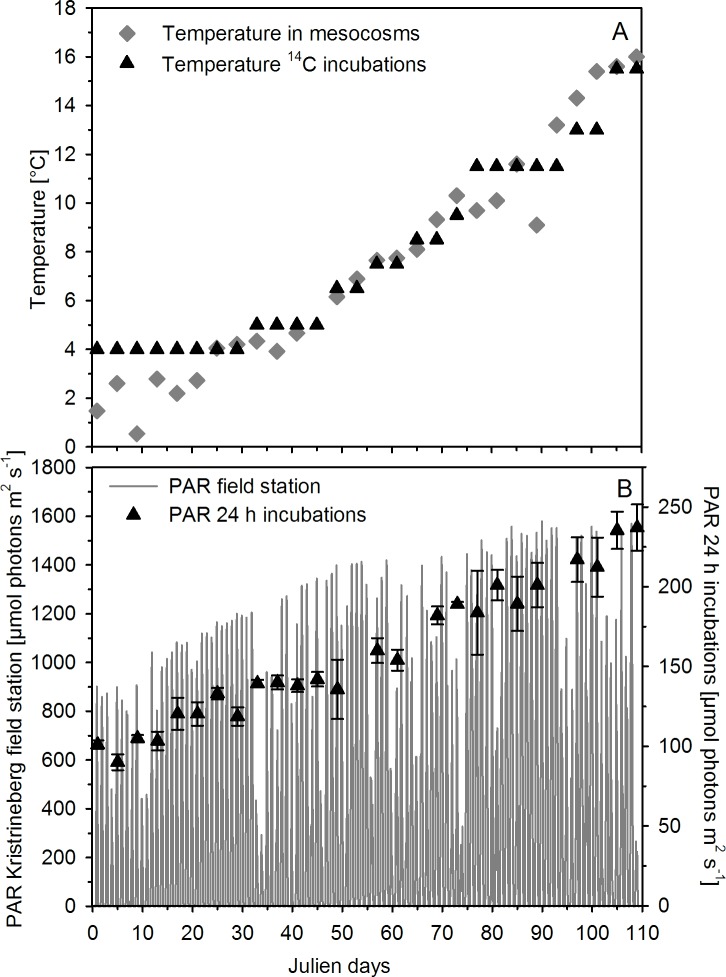
Mean temperature in mesocosms (grey diamonds) and during ^14^C incubations (black triangles) (A), and incoming light (PAR) at the Kristineberg field station around midday (http://www.weather.loven.gu.se/kristineberg/en; grey lines) and during ^14^C-based 24 h incubations (black triangles) (B). Triangles indicate the mean ± SD of three light measurements from the bottom, middle and top of a representative incubation vial.

For our measurements, integrated water samples from 0–17 m depth were taken in a four day interval (i.e. t1, t5, t9, etc.) from each of the ten mesocosms, and an additional sample was taken from the fjord. Sampling usually took place between 9 and 12 a.m. and aliquots from well mixed water samples were filled in gas-tight and headspace-free bottles (Schott) of 250 mL (for the 24 h incubations) and 500 mL (for the photosynthesis-irradiance response (P/I) curves). Samples were brought directly to the laboratory, where they were gently filtered over a 500 μm mesh-size filter to remove larger zooplankton from the samples, and were kept at the *in situ* water temperature until incubations started. Over the course of the entire experiment, the temperature in the fjord increased from 1.5°C at t1 towards 15.5°C at t109, and we adjusted the incubation temperatures accordingly (Fig 1A). Only at the beginning of the experiment, when productivity and biomass was still low, we could not fully match the temperature from the fjord as our incubator was not able to maintain temperatures below 4°C. Light was provided by daylight tubes (OSRAM) from the side in a 16:8 h light-dark cycle. To account for the increase in light intensities over the course of the experiment in the mesocosms, the light intensity was stepwise increased in the incubator (Fig 1B). Using a spherical micro quantum sensor (Walz), we increased the photon flux density (PFD) every 16 days (i.e. after 4 sampling days) by about 20 μmol photons m^-2^ s^-1^, starting with around 100 μmol photons m^-2^ s^-1^ at t1 and ending with 240 μmol photons m^-2^ s^-1^ at t109.

### Primary production measurements

Primary productivity was measured according to Steeman Nielsen [24]. Despite limitations [27], this approach has remained the method of choice, especially for field work, as it allows assessing rates even at times of low productivity. One has to keep in mind, however, that measured rates have different meanings depending on the incubation time [27]. In our 80 min. incubations for ^14^C-based photosynthesis-irradiance response (P/I) curves, we obtained rates of gross primary production because there is only little loss of incorporated ^14^C via respiration and exudation over such short timescales. In our 24 h incubations for the ^14^C-based primary production measurements, respiration lowers the ^14^C incorporation and thus net rates of primary production are obtained. To account for fixed ^14^C ending up in the dissolved phase, which can be a significant proportion under nutrient deplete conditions, we included values of the filtrate in our PP_T_ estimates.

### ^14^C-based primary production measurements

For the 24 h incubations, 40 mL sample volumes were spiked with 20 μL of ^14^C-labeled sodium bicarbonate (NaH^14^CO_3_; from a 1 mCi mL^-1^ = 37 MBq mL^-1^ stock solution; PerkinElmer). Two incubation vials for each mesocosm, and the fjord water, were prepared accordingly (i.e. 22 vials in total, of which 11 were used for the light and the remaining 11 for the dark incubations). Determination of total ^14^C-spike addition was done from an extra 40 mL ^14^C-spiked water sample. For this purpose, 1 mL was directly transferred into a 20 mL scintillation vial (PerkinElmer) containing 10 mL scintillation cocktail (Ultima Gold AB; PerkinElmer) and counted in a liquid scintillation analyzer (Beckman LS6500). Blank determination was done by transferring 1 mL from the extra ^14^C-spiked water sample into 6 mL of 6 M HCl, which degassed for 48 h and was then counted after adding 10 mL scintillation cocktail. All incubations were placed on an orbital shaker in a temperature-controlled incubator.

Incubations were stopped after 24 h by vacuum filtration onto GF/F filters (Whatman). To estimate the amount of C_i_ fixation into particulate organic carbon (POC), filters were rinsed twice with 20 mL of sterile filtered seawater (0.2 μm), and subsequently placed in scintillation vials containing 300 μL of 3 M HCl to remove ^14^C-labeled DIC. To estimate the amount of C_i_ fixation ending up in the pool of dissolved organic carbon (DOC), 6 mL of filtrate was transferred into a scintillation vial, acidified with 1 mL 6 M HCl, and placed under a fume hood for 48 hours for degassing DI^14^C. Prior to measurements, 10 mL of scintillation cocktail was added to each vial and filter, thoroughly mixed, and counted in a liquid scintillation analyzer. Primary production (PP) was calculated according to:

$$PP=\frac{DIC∙({DPM}_{sample}-{DPM}_{blank})∙1.05}{\left({DPM}_{100\%}∙t\right)}$$ Eq 1

where *DPM* represents the decays per minute and *t* represents time. Correction for non-specific ^14^C fixation in the dark was done by subtracting dark incubations from light incubations. Dark ^14^C fixation accounted for about 1 to 6% of the light incubations during times of high and low productivity, respectively. Based on the phytoplankton community composition [21], some primary producers were smaller than the pore size of our filters (i.e. <0.7 μm). We therefore reported total primary production (PP_T_; μmol C L^-1^ h^-1^) from the 24 h incubations as the sum of CO_2_ fixation into POC and DOC.

### ^14^C-based photosynthesis-irradiance response curves

For the photosynthesis-irradiance response (P/I) curves, 300 mL sample volume from each mesocosm was spiked with 100 μCi of NaH^14^CO_3_^-^ (PerkinElmer) and subdivided into seven 40 mL glass vials. From the remaining ^14^C-spiked seawater, 200 μL aliquots were transferred into a 10 mL scintillation cocktail to determine total spike addition for each P/I curve. While one vial was incubated in the dark, the six remaining vials were exposed to increased light intensities ranging from about 10 to 700 μmol photons m^-2^ s^-1^ in a custom-made photosynthetron. Light was supplied from below and the PFD was assessed prior to each experimental day. The photosynthetron was placed in the same incubator as the 24 h incubations. Additional temperature control was achieved via a water bath connected to the sample holder. After an incubation time of 80 min. at the respective light conditions, samples were filtered on GF/F filters (Whatman). Analysis of PO^14^C was determined following the same procedure as for the 24 h incubations and data was fitted according to:

$${PP}_{P/I}=P_{max}∙(1-e^{-\alpha ∙(I-I_{k})})$$ Eq 2

where *P*_*max*_ is the light-saturated rate of photosynthesis, *α* is the light-limited (i.e. initial) slope of the P/I curve representing the maximum light-use efficiency, *I* is the irradiance, and *I*_*k*_ is the light saturation index. Rates of PP_P/I_ were normalized to chlorophyll *a* (Chl *a*) concentrations in the samples from the particular day and mesocosm [21].

### Statistics

Differences in PP_T_, Chl *a*, P_*max*_, *I*_*k*_ and α between the CO_2_ treatments were tested over time by a two-way repeated measures Analysis of Variance (rmANOVA), and the association between PP_T_ and Chl *a* was tested by Pearson product-moment correlations. Variables were log+1 or square root transformed if this improved normality or homogeneity of variances, as tested by the Shapiro-Wilk test or Levene’s test, respectively. All statistics were performed with Sigmaplot 12.5 (Systat).

## Results

### Total primary production

**Fig 2:**
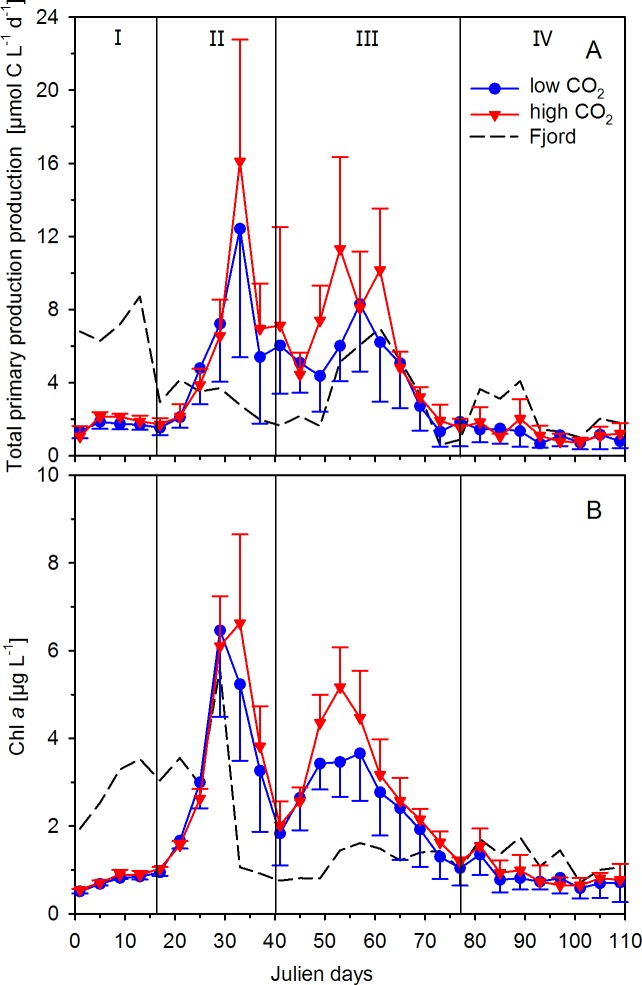
Mean values of total primary production (from ^14^C-based 24 h incubations; A) and chlorophyll *a* concentrations (B) from mesocosm and fjord samples. Triangles (red; high *p*CO_2_) and circles (blue; low *p*CO_2_) represent the mean ± SD of five biological replicates. Roman numbers denote the different phases of the experiment.

For the first three weeks of the experiment, estimates on PP_T_ were lower in the mesocosms than in the fjord (Fig 2A). All mesocosms showed comparable development in PP_T_, with an initial period of low productivity (phase I, t1-t16), a first spring bloom of highest productivity around t33 (phase II, t17-t40), followed by a second bloom of highest productivity around t57 (phase III, t41-77), and a subsequent period of low productivity until the end of the experiment (phase IV, t78-t109; Fig 2A, Table 1). Dynamics in primary production in the mesocosms differed from that in the fjord. For example, PP_T_ was higher in the fjord during phase I, while PP_T_ was higher in the mesocosms during phase II. Also, a small increase in PP_T_ present in the fjord at the start of phase IV was lacking in the mesocosms (Fig 2A).

**Table 1:** Total primary production (μmol C L^-1^ d^-1^) in the mesocosms derived from 24 h incubations. Values at high *p*CO_2_ are indicated in bold letters (M2, M4, M6-8). Grey shading indicates the peak of the two bloom phases.

Julien day	M1	M2	M3	M4	M5	M6	M7	M8	M9	M10
1	1.88	**1.34**	0.85	**1.42**	1.44	**1.46**	**0.95**	**0.08**	1.16	1.28
5	1.75	**1.97**	1.29	**2.04**	2.15	**2.58**	**2.12**	**1.96**	1.82	2.26
9	1.96	**2.16**	1.25	**2.30**	1.93	**2.27**	**1.96**	**2.01**	1.93	1.65
13	1.69	**1.60**	1.26	**2.22**	2.02	**2.22**	**1.81**	**1.57**	1.80	1.68
17	1.48	**1.53**	1.83	**1.48**	0.85	**1.43**	**2.13**	**2.03**	1.88	1.61
21	2.63	**2.15**	2.04	**2.46**	1.77	**3.07**	**1.34**	**1.66**	1.34	2.72
25	6.08	**4.60**	2.81	**4.22**	2.65	**2.92**	**4.70**	**2.98**	5.34	7.05
29	9.48	**6.88**	4.68	**8.39**	3.27	**6.45**	**7.77**	**3.30**	7.88	10.79
33	15.94	**5.04**	4.92	**22.40**	13.20	**15.47**	**18.89**	**18.73**	6.16	21.83
37	7.70	**3.40**	3.29	**9.70**	4.05	**7.62**	**8.37**	**5.65**	1.45	10.47
41	8.91	**3.08**	5.25	**16.59**	2.02	**5.11**	**5.17**	**5.68**	6.32	7.65
45	5.10	**2.73**	6.98	**5.25**	2.50	**5.48**	**5.07**	**3.66**	5.14	5.70
49	5.65	**8.65**	2.45	**8.10**	3.43	**8.60**	**4.10**	**7.59**	3.16	7.15
53	9.01	**4.90**	4.93	**14.08**	5.80	**12.51**	**17.43**	**7.65**	3.89	6.41
57	9.69	**6.15**	3.91	**9.96**	9.95	**10.87**	**9.84**	**3.59**	12.80	5.10
61	6.77	**7.60**	2.78	**13.59**	3.42	**6.64**	**13.83**	**9.15**	7.24	10.85
65	5.77	**4.71**	1.24	**4.10**	4.49	**6.21**	**4.98**	**4.08**	5.90	7.90
69	3.13	**2.54**	1.06	**3.80**	1.85	**2.83**	**3.69**	**3.17**	4.55	2.97
73	1.05	**1.75**	0.69	**1.71**	0.65	**1.84**	**3.36**	**0.92**	1.58	2.62
77	3.68	**0.90**	0.85	**1.94**	0.53	**1.97**	**1.70**	**1.22**	1.41	2.77
81	2.40	**0.39**	0.72	**2.05**	0.86	**1.93**	**2.41**	**2.40**	1.36	1.79
85	2.16	**1.28**	1.35	**0.88**	0.75	**0.98**	**1.07**	**1.01**	0.65	2.49
89	1.10	**1.13**	1.02	**1.52**	1.08	**1.40**	**3.74**	**2.37**	0.67	2.83
93	0.29	**0.52**	0.73	**1.23**	0.81	**0.80**	**1.98**	**0.94**	0.83	0.65
97	1.29	**0.57**	1.01	**0.43**	0.19	**0.47**	**1.44**	**0.98**	1.27	1.79
101	0.83	**0.63**	0.41	**1.02**	1.30	**0.64**	**0.44**	**0.95**	0.50	0.52
105	0.78	**0.85**	1.91	**0.99**	2.11	**1.98**	**0.86**	**0.85**	0.35	0.62
109	1.05	**1.65**	0.48	**0.65**	1.34	**0.54**	**1.63**	**1.60**	0.48	0.66

High *p*CO_2_ yielded higher mean estimates on PP_T_ during both blooms, although differences during both blooms were not significant (Table 2). Highest PP_T_ was observed during the first bloom at t33, with up to 16.1 ± 6.7 μmol C L^-1^ d^-1^ at high *p*CO_2_ and 12.4 ± 7.0 μmol C L^-1^ d^-1^ at low *p*CO_2_. During the second bloom, PP_T_ amounted to highest values of 11.3 ± 5.0 μmol C L^-1^ d^-1^ at t53 for high *p*CO_2_, and 6.0 ± 1.9 μmol C L^-1^ d^-1^ at day t57 for low *p*CO_2_ At the peak of the second bloom, PP_T_ appeared to be higher at high *p*CO_2_, though this difference was marginally significant and dependent on time (Table 2; rmANOVA, Time x CO_2_ treatment, P = 0.098). During both blooms phases, Chl *a* remained unaltered in response to OA (Table 2), though at times showed higher concentrations at high *p*CO_2_ [21]. Furthermore, Chl *a* was strongly correlated to PP_T_ (σ = 0.87, P < 0.0001).

**Table 2:** Output of the repeated measures ANOVA for phase II, phase III, peak of bloom 1 and peak of bloom 2, with degrees of freedom (df), the *F*-value and the *P*-value. Significant outcomes are indicated with P < 0.001 (***), P < 0.01 (**), P < 0.05 (*) and P < 0.1 (**∙**).

	Parameter	Effect	df	*F*	*P*
Phase II (incl. bloom 1; t17-t40)	PP_T_ (μmol C L^-1^ d^-1^)	CO_2_ treatment	1	0.278	0.612
		Time	5	38.060	<0.001***
		Time x CO_2_ treatment	5	0.814	0.547
	Chl *a* (μg Chl *a* L^-1^)	CO_2_ treatment	1	0.228	0.646
		Time	5	13.818	<0.001***
		Time x CO_2_ treatment	5	0.245	0.940
	P_max_ (μg C (μg Chl *a*)^-1^ h^-1^)	CO_2_ treatment	1	0.845	0.383
		Time	5	15.796	<0.001***
		Time x CO_2_ treatment	5	0.497	0.776
	*I*_K_ (μmol photons m^-2^ s^-1^)	CO_2_ treatment	1	0.651	0.443
		Time	5	2.647	0.037*
		Time x CO_2_ treatment	5	0.712	0.618
	Alpha	CO_2_ treatment	1	0.023	0.883
		Time	5	10.814	<0.001***
		Time x CO_2_ treatment	5	0.633	0.676
Phase III (incl. bloom 2; t41-t77)	PP_T_ (μmol C L^-1^ d^-1^)	CO_2_ treatment	1	1.481	0.258
		Time	9	26.124	<0.001***
		Time x CO_2_ treatment	9	1.566	0.142
	Chl *a* (μg Chl *a* L^-1^)	CO_2_ treatment	1	0.395	0.547
		Time	9	9.258	<0.001***
		Time x CO_2_ treatment	9	0.915	0.517
	P_max_ (μg C (μg Chl *a*)^-1^ h^-1^)	CO_2_ treatment	1	1.538	0.250
		Time	9	4.126	<0.001***
		Time x CO_2_ treatment	9	0.565	0.821
	*I*_K_ (μmol photons m^-2^ s^-1^)	CO_2_ treatment	1	0.181	0.681
		Time	9	4.544	<0.001***
		Time x CO_2_ treatment	9	0.422	0.919
	Alpha	CO_2_ treatment	1	0.767	0.407
		Time	9	6.914	<0.001***
		Time x CO_2_ treatment	9	1.338	0.233
Peak of bloom 1 (t29-t33)	PP_T_ (μmol C L^-1^ d^-1^)	CO_2_ treatment	1	0.340	0.576
		Time	1	13.682	0.006**
		Time x CO_2_ treatment	1	1.194	0.306
	Chl *a* (μg Chl *a* L^-1^)	CO_2_ treatment	1	0.092	0.769
		Time	1	50.114	<0.001***
		Time x CO_2_ treatment	1	0.152	0.707
	P_max_ (μg C (μg Chl *a*)^-1^ h^-1^)	CO_2_ treatment	1	0.372	0.559
		Time	1	38.768	<0.001***
		Time x CO_2_ treatment	1	0.017	0.899
	*I*_K_ (μmol photons m^-2^ s^-1^)	CO_2_ treatment	1	0.858	0.381
		Time	1	0.590	0.465
		Time x CO_2_ treatment	1	0.581	0.468
	Alpha	CO_2_ treatment	1	0.360	0.565
		Time	1	33.248	<0.001***
		Time x CO_2_ treatment	1	0.653	0.442
Peak of bloom 2 (t53-t61)	PP_T_ (μmol C L^-1^ d^-1^)	CO_2_ treatment	1	3.134	0.115
		Time	2	0.099	0.907
		Time x CO_2_ treatment	2	2.701	0.098∙
	Chl *a* (μg Chl *a* L^-1^)	CO_2_ treatment	1	1.200	0.305
		Time	2	2.168	0.147
		Time x CO_2_ treatment	2	2.278	0.135
	P_max_ (μg C (μg Chl *a*)^-1^ h^-1^)	CO_2_ treatment	1	0.506	0.497
		Time	2	9.666	0.002**
		Time x CO_2_ treatment	2	0.029	0.972
	*I*_K_ (μmol photons m^-2^ s^-1^)	CO_2_ treatment	1	0.120	0.738
		Time	2	5.210	0.018**
		Time x CO_2_ treatment	2	1.874	0.186
	Alpha	CO_2_ treatment	1	0.072	0.795
		Time	2	1.366	0.283
		Time x CO_2_ treatment	2	0.311	0.737

**Fig 3:**
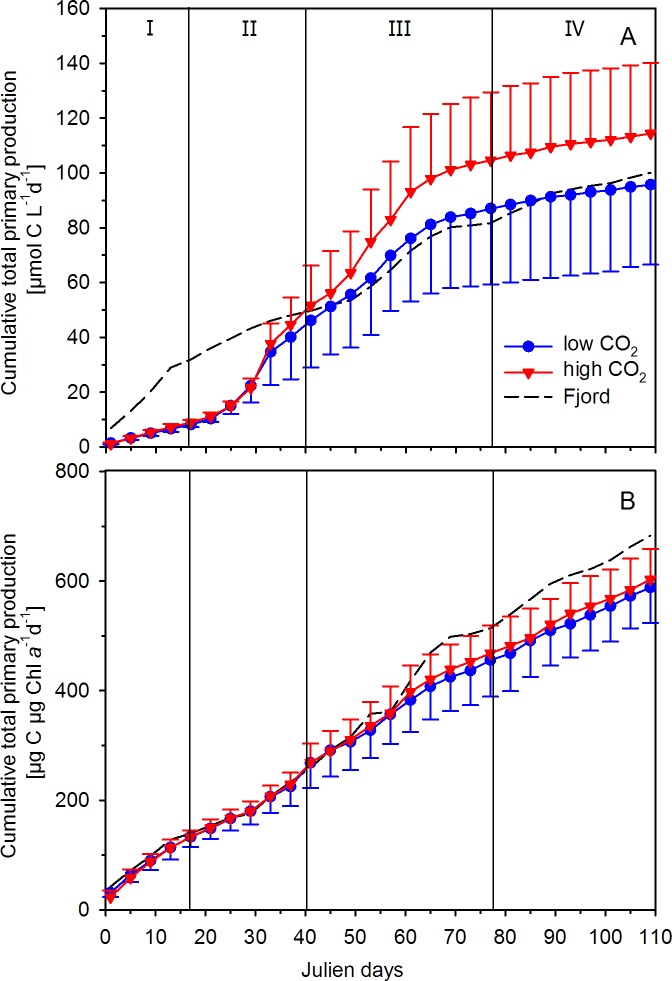
Cumulative total primary production (from ^14^C-based 24 h incubations; A) and normalized to chlorophyll *a* concentrations (B) from mesocosm and fjord samples. Triangles (red; high *p*CO_2_) and circles (blue; low *p*CO_2_) represent the mean ± SD of five biological replicates. Roman numbers denote the different phases of the experiment.

When cumulated over the experimental period of 109 days, the PP_T_ data yielded a total of 92 ± 29.21 and 110 ± 25.79 μmol C L^-1^ at low and high *p*CO_2_, respectively. In the fjord, cumulative PP_T_ yielded 95 μmol C L^-1^ (Fig 3A), being more comparable to PP_T_ in the mesocosms at low *p*CO_2_. The difference in cumulative PP_T_ between low and high *p*CO_2_ was about 20% and closely matched the observed difference in Chl *a* concentration of about 15%. Consequently, no differences in the yields were observed when normalizing cumulated PP_T_ to Chl *a* (as to account for changes in phytoplankton biomass). In both treatments, we got a cumulative value of around 600 μg C (μg Chl *a*)^-1^ until the end of the experiment (Fig 3B). Chl *a*-normalized cumulative PP_T_ in the fjord was higher than in the mesocosms and amounted to a total of about 700 μg C (μg Chl *a*)^-1^ (Fig 3B).

### Photoacclimation

**Fig 4:**
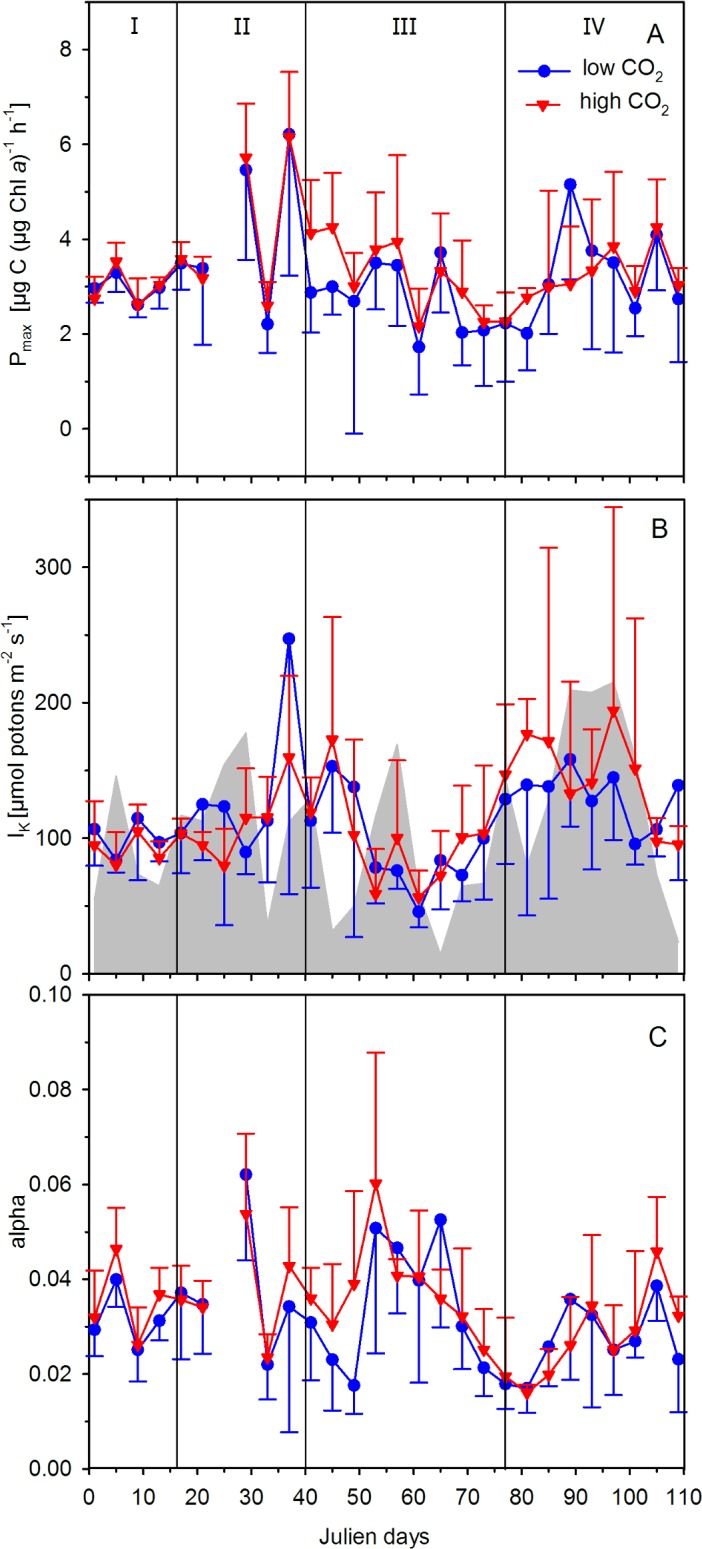
Light-saturated maximum rates (*P*_*max*_; A), light saturation index (*I*_*k*_; B), and light-limited slope (alpha; C) of the photosynthesis-response irradiance curves. Triangles (high *p*CO_2_) and circles (low *p*CO_2_) represent the mean ± SD of five biological replicates. The grey area in Fig B indicates average water column light intensities (0–19 m depth) during midday for all ten mesocosms. Roman numbers denote the different phases of the experiment.

P/I curves provided information on the photoacclimation of the phytoplankton communities in the mesocosms and the fjord. *P*_*max*_ was on average 3.17 ± 0.54 and 3.38 ± 0.26 μg C (μg Chl *a*)^-1^ h^-1^ at low and high *p*CO_2_, respectively. There was no apparent CO_2_ effect on *P*_*max*_ during both blooms (Table 2), which furthermore strongly varied between mesocosms and sampling days (Fig 4A). *I*_*k*_, indicating the light intensity at which phytoplankton shifts from light limitation to light saturation, changed over the course of the experiment (Fig 4B). More specifically, in the period prior to the first bloom (phase I), *I*_*k*_ remained around 100 μmol photons m^-2^ s^-1^ and increased towards the end of the first bloom phase reaching mean values of approximately 160 and 250 μmol photons m^-2^ s^-1^ at high and low *p*CO_2_, respectively. In the course of the second bloom, *I*_*k*_ decreased resulting in lowest values of 50 μmol photons m^-2^ s^-1^ around t61 (Fig 4), after which it increased again to values of around 150 μmol photons m^-2^ s^-1^ (Fig 4B). Besides these general changes over the season, we did not observe a significant CO_2_ effect on *I*_k_ values during both blooms (Table 2). The maximum light-use efficiency also changed in the course of the phytoplankton succession. Highest α values coincided with the phytoplankton blooms during phases II and III and were observed around t30 and t56 in all mesocosms (Fig 4C). Similar to the other parameters, there was no significant CO_2_ effect on *α* values during both blooms (Table 2).

## Discussion

We did not observe a sustained effect of OA on primary production during the investigated winter-to-summer plankton succession. When focusing on the peak of the second spring bloom in phase III, however, PP_T_ showed a marginally significant increase under high *p*CO_2_ (Table 2). During this distinct phase, the availability of inorganic nutrients was low and primary production was fueled by *in situ* remineralization [21]. Integrated over the entire experimental period, OA yielded about 20% more CO_2_ fixation. Such enhanced primary production is in line with the higher Chl *a* concentration under these conditions.

At the onset of the experiment, concentrations of major nutrients in the mesocosms were higher than in the fjord [21]. The lower concentrations in the fjord were the result of higher primary production compared to the mesocosms right after closure of the mesocosms (beginning at t-2, Fig 2). Although initial conditions in the mesocosms were largely comparable to the situation in the fjord, perturbations induced during the set-up of the mesocosms [21], e.g. the water column mixing (t0) or the establishment of CO_2_ treatments (t-1 and t0), may have contributed to the delay in primary production.

In the mesocosms, PP_T_ as well as Chl *a* concentrations remained relatively low during phase I and started to increase more pronounced around t20, leading to a first phytoplankton bloom with highest PP_T_ around t33 (Fig 2). During this phase II, major nutrients such as inorganic phosphate and nitrogen were depleted to very low values (for more details, see Bach et al. [21]). This nutrient depletion, particularly for nitrogen, together with grazing presumably caused the collapse of the phytoplankton bloom and the decrease in PP_T_ as well as Chl *a* concentrations (Fig 2). At the same time at the Kristineberg field station (~3 km distance to mesocosm deployment site), a sudden drop in the *in situ* light intensity was detected (Fig 1B), coinciding with the peak of the first bloom (Fig 2A). In fact, the average water column light intensities (0–19 m depth) during midday for all ten mesocosms were reduced to about 35 μmol photons m^−2^ s^−1^ for several days (Fig 4B), and dropped below the *I*_*k*_ values (about 115 μmol photons m^−2^ s^−1^). Such lower light levels may possibly have, at least temporally, limited photosynthesis and thereby affected the response of phytoplankton to low nutrient levels. While there were dynamic changes in *P*_*max*_, *I*_*k*_, and *α* over the course of the phytoplankton succession (Fig 4), there was no effect of OA on photoacclimation.

Dissolved phosphate and inorganic nitrogen concentrations remained low during phase III, while PP_T_ and Chl *a* concentrations increased again, causing the second bloom (Fig 2). An earlier study in the Gullmar Fjord also showed a relatively high primary production during summer months, despite low nutrient concentrations [28,29]. According to this long-term Gullmar Fjord time-series study, nutrients were not only derived from recycled production, but also from local precipitation, run-off, and input from the Kattegat [29]. As the mesocosms were isolated from the surrounding water, nutrient input for primary production should have derived from regeneration only. In fact, dissolved organic nitrogen and NH_4_^+^ concentrations in the mesocosms remained low, indicating a rapid cycling of nutrients in the food web [21]. Interestingly, it is /under these conditions of recycled production and low concentrations of inorganic nutrients that we observed the strongest response in PP_T_ towards OA (Fig 2A). Under nutrient-limited conditions, effects of elevated *p*CO_2_ on phytoplankton productivity, standing stock and community composition were often found to be stronger [30–32] and comparable findings were also reported with respect to iron limitation [33]. Since nitrogen, phosphorus and iron predominantly limit phytoplankton growth in the global surface oceans [34,35], more studies investigating the combined effects of elevated *p*CO_2_ and resource limitation are required to provide a mechanistic understanding on the impacts of OA on future primary production.

Even though we did not find a consistent CO_2_ response over the entire winter-to-summer plankton succession, the stimulation in primary production under elevated *p*CO_2_ at the peak of the second bloom was also observed in earlier studies looking at mixed natural assemblages as well as monoclonal laboratory cultures. During a mesocosm study in Bergen (Norway), for instance, DIC uptake increased under a comparable OA scenario by about 40% [30,36]. Moreover, a mesocosm study in Kongsfjorden (Svalbard, Norway) showed an OA-induced increase in primary production of 10 to 60% over the experimental period [37]. Such increases in primary production may derive from physiological changes in predominant species and/or shifts in community composition both leading to higher phytoplankton biomass buildup. At a higher taxonomic level, the phytoplankton community remained largely unaltered and was dominated by diatoms [21]. Under nutrient-replete as well as nutrient-limiting conditions, elevated *p*CO_2_ resulted in an increased abundance of picoeukaryotes [21]. Specific changes within phytoplankton groups will be discussed elsewhere in this special issue (see S1 Table in [21]). With regard to the dominating role of diatoms in our experiment, several studies found this group to enhance their C_i_ fixation rates in response to elevated *p*CO_2_, which was often attributed to the down-regulation in the CCM activities under these conditions (e.g. [38–40]). Such enhanced OA-driven efficiencies in C_i_ fixation may, at least partially, have contributed to the higher phytoplankton biomass during the second bloom in our experiment.

Our results indicate an OA-dependent increase in primary production during certain times of the spring-to-summer phytoplankton succession, particularly under NO_3_^-^ limitation (phase III) being accompanied by a significant increase in picoeukaryotes during this period [21]. With respect to higher trophic levels, OA showed differential growth effects on several predominant mesozooplankton species, though as a whole, the community remained rather unaltered under OA (Alguero et al. *in prep*.). OA led, however, to an increase in the survival rate of herring larvae (being planted in the mesocosms on t63), which could be linked to higher prey abundances (Swaat et al. *in prep*.). Hence, the observed changes in primary production under OA have a high potential to restructure phytoplankton communities in the future coastal North Sea with likely consequences for higher trophic levels.

## Supporting information

S1 Fig PAR (μmol m^-2^ s^-1^) inside the mesocosms during the time of the experiment. (PNG)

S1 Table Raw data of photoacclimation parameters from photosynthesis-irradiance response curves. (XLSX)

## References

[1] Wolf-Gladrow DA, Riebesell U, Burkhardt S, Bijma J. Direct effects of CO_2_ concentration on growth and isotopic composition of marine plankton. Tellus B. 1999; 51: 461–476.

[2] Caldeira K, Wickett ME. Anthropogenic carbon and ocean pH. Nature. 2003; 425: 365 10.1038/425365a 14508477

[3] Stocker TF, Qin D, Plattner G-K, Tignor M, Allen SK, Boschung J, et al (eds.). 2013 In: climate change 2013: the physical science basis Contribution of working group I to the fifth assessment report of the intergovernmental panel on climate change. Cambridge University Press, USA.

[4] Roy T, Bopp L, Gehlen M, Schneider B, Cadule P, Frölicher TL, et al Regional impacts of climate change and atmospheric CO_2_ on future ocean carbon uptake: A multimodel linear feedback analysis. J. Clim. 2011; 24: 2300–2318.

[5] Sabine CL, Feely RA, Gruber N, Key RM, Lee K, Bullister JL, et al The oceanic sink for anthropogenic CO_2_. Science. 2004; 305: 367–371. 10.1126/science.1097403 15256665

[6] Raven JA. Inorganic carbon acquisition by marine autotrophs. Adv. Bot. Res. 1997; 27: 85–209.

[7] Badger MR, Andrews TJ, Whitney SM, Ludwig M, Yellowlees DC, Leggat W, Price GD. The diversity and coevolution of Rubisco, plastids, pyrenoids, and chloroplast-based CO_2_-concentrating mechanisms in algae. Can. J. Bot. 1998; 76: 1052–1071.

[8] Young JN, Heureux AMC, Sharwood RE, Rickaby REM, Morel FMM, Whitney SM. Large variation in the Rubisco kinetics of diatoms reveals diversity among their carbon-concentrating mechanisms. J. Exp. Bot. 2016; 10.1093/jxb/erw163 PMC4892730 27129950

[9] Raven JA. Physiology of inorganic C acquisition and implications for resource use efficiency by marine phytoplankton: relation to increased CO_2_ and temperature. Plant Cell Environ. 1991; 14: 779–794.

[10] Giordano M, Beardall J, Raven JA. CO_2_ concentrating mechanisms in algae: mechanisms, environmental modulation, and evolution. Annu. Rev. Plant. Biol. 2005; 56: 99–131. 10.1146/annurev.arplant.56.032604.144052 15862091

[11] Rost B, Zondervan I, Wolf-Gladrow D. Sensitivity of phytoplankton to future changes in ocean carbonate chemistry: current knowledge, contradictions and research directions. Mar. Ecol. Prog. Ser. 2008; 373: 227–237.

[12] Reinfelder JR. Carbon concentrating mechanisms in eukaryotic marine phytoplankton. Annu. Rev. Mar. Sci. 2011; 3: 291–315. 10.1146/annurev-marine-120709-142720 21329207

[13] Raven JA, Beardall J, Giordano M. Energy costs of carbon dioxide concentrating mechanisms in aquatic organisms. Photosynth Res. 2014; 121(2–3):111–24. 10.1007/s11120-013-9962-7 24390639

[14] Burkhardt S, Zondervan I, Riebesell U. Effect of CO_2_ concentration on C:N:P ratio in marine phytoplankton: a species comparison. Limnol. Oceanogr. 1999; 44: 683–690.

[15] Kranz SA, Sültemeyer D, Richter K-U, Rost B. Carbon acquisition by *Trichodesmium*: the effect of *p*CO_2_ and diurnal changes. Limnol. Oceanogr. 2009; 54: 548–559.

[16] Eberlein T, Van de Waal DB, Brandenburg KM, John U, Voss M, Achterberg EP, et al Interactive effects of ocean acidification and nitrogen limitation on two bloom-forming dinoflagellate species. Mar. Ecol. Prog. Ser. 2016; 543: 127–140.

[17] Hutchins DA, Fu F-X, Zhang Y, Warner ME, Feng Y, Portune K, et al CO_2_ control of *Trichodesmium* N_2_ fixation, photosynthesis, growth rates, and elemental ratios: Implications for past, present, and future ocean biogeochemistry. Limnol. Oceanogr. 2007; 52(4): 1293–1304.

[18] Brading P, Warner ME, Smith DJ, Suggett DJ. Contrasting modes of inorganic carbon acquisition amongst *Symbiodinium* (Dinophyceae) phylotypes. New Phytol. 2013; 200: 432−442. 10.1111/nph.12379 23815769

[19] Schaum E, Rost B, Millar AJ, Collins S. Variation in plastic responses of a globally distributed picoplankton species to ocean acidification. Nat. Clim. Change. 2013; 3: 298–302.

[20] Riebesell U, Tortell PD. Effects of ocean acidification on pelagic organisms and ecosystems In: Gattuse J.P., Hansson L. (Eds.), Ocean Acidification. Oxford University Press, Oxford, UK, 2011; 99–121.

[21] Bach LT, Taucher J, Boxhammer T, Ludwig A, The Kristineberg KOSMOS Consortium, Achterberg EP, et al Influence of Ocean Acidification on a Natural Winter-to-Summer Plankton Succession: First Insights from a Long-Term Mesocosm Study Draw Attention to Periods of Low Nutrient Concentrations. PLoS ONE. 2016; 11(8). e0159068 10.1371/journal.pone.0159068 27525979 PMC4985126

[22] Tiselius P, Kuylenstierna M. Growth and decline of a diatom spring bloom: phytoplankton species composition, formation of marine snow and the role of heterotrophic dinoflagellates. J. Plankton. Res. 1996; 18: 133–155.

[23] Waite AM, Lindahl O. Bloom and decline of the toxic flagellate *Chattonella marina* in a Swedish fjord. Mar. Ecol. Prog. Ser. 2006; 326: 77–83.

[24] Steemann Nielsen E. The use of radioactive carbon (^14^C) for measuring primary production in the sea. J. Cons. Perm. Int. Explor. Mer. 1952; 18: 117–140.

[25] Riebesell U, Czerny J, Bröckel KV, Boxhammer T, Büdenbender J, Deckelnick M, et al Technical Note: A mobile sea-going mesocosm system–new opportunities for ocean change research. Biogeosciences. 2013; 10(3): 1835–1847.

[26] Boxhammer T, Bach LT, Czerny J, Riebesell U. Technical note: Sampling and processing of mesocosm sediment trap material for quantitative biogeochemical analysis. Biogeosciences. 2016; 13: 2849–2858.

[27] Bender M, Grande K, Johnson K, Marra J, Williams PJ LeB, Sieburth J, et al A comparison of four methods for determining planktonic community production. Limnol. Oceanogr. 1987; 32(5): 1085–1098.

[28] Lindahl O. Long-term studies of primary production in the Gullmar fjord, Sweden *In*: Skjoldal H. R., Hopkins C., Erikstad K. E., P Leinaas H. [eds.] Ecology of fjords and coastal waters. Elsevier Science Publishers, New York 1995; 105–112.

[29] Lindahl O, Belgrano A, Davidsson L, Hernroth B. Primary production, climatic oscillations, and physico-chemical processes: the Gullmar Fjord time-series data set (1985–1996). ICES J. Mar. Sci. 1998; 55 (4): 723–729.

[30] Egge JK, Thingstad TF, Larsen A, Engel A, Wohlers J, Bellerby RGJ, et al Primary production during nutrient-induced blooms at elevated CO_2_ concentrations. Biogeosciences. 2009; 6: 877–885.

[31] Paul AJ, Bach LT, Schulz KG, Boxhammer T, Czerny J, Achterberg EP, et al Effect of elevated CO_2_ on organic matter pools and fluxes in a summer Baltic Sea plankton community. Biogeosciences. 2015; 12: 6181–6203.

[32] Sala MM, Aparicio FL, Balagué V, Boras JA, Borrull E, Cardelús C. Contrasting effects of ocean acidification on the microbial food web under different trophic conditions. Ices. J. Mar. Sci. 2015;

[33] Hopkinson BM, Xu Y, Shi D, McGinn PJ, Morel FMM. The effect of CO_2_ on the photosynthetic physiology of phytoplankton in the Gulf of Alaska. Limnol. Oceanogr. 2010; 55(5): 2011–2024.

[34] Elser JJ, Bracken MES, Cleland EE, Gruner DS, Harpole WS, Hillebrand H, et al Global analysis of nitrogen and phosphorus limitation of primary producers in freshwater, marine and terrestrial ecosystems. Ecol. Lett. 2007; 10: 1135−1142. 10.1111/j.1461-0248.2007.01113.x 17922835

[35] Moore CM, Mills MM, Arrigo KR, Berman-Frank I, Bopp L, Boyd PW, et al Processes and patterns of oceanic nutrient limitation. Nat. Geosci. 2013; 6: 701−710.

[36] Riebesell U, Schulz KG, Bellerby RGJ, Botros M, Fritsche P, Meyerhofer M, et al Enhanced biological carbon consumption in a high CO_2_ ocean. Nature. 2007; 450: 545–548. 10.1038/nature06267 17994008

[37] Engel A, Borchard C, Piontek J, Schulz KG, Riebesell U, Bellerby R. CO_2_ increases ^14^C primary production in an Arctic plankton community. Biogeosciences. 2013; 10: 1291–1308.

[38] Trimborn S, Lundholm N, Thoms S, Richter K-U, Krock B, Hansen PJ et al Inorganic carbon acquisition in potentially toxic and non-toxic diatoms: the effect of pH-induced changes in seawater carbonate chemistry. Physiol. Plant. 2008; 133: 92–105. 10.1111/j.1399-3054.2007.01038.x 18405335

[39] Wu Y, Gao K, Riebesell U. CO_2_-induced seawater acidification affects physiological performance of the marine diatom *Phaeodactylum tricornutum*. Biogeosciences. 2010; 7: 2915–2923.

[40] Hoppe CJM, Holtz L-M, Trimborn S, Rost B. Ocean Acidification decreases the light use efficiency in an Antarctic diatom under dynamic but not constant light, New Phytol. 2015; 207: 159–171. 10.1111/nph.13334 25708812 PMC4950296

