# Supplementary figures and images for: Effects of ocean acidification on primary production in a coastal North Sea phytoplankton community
Source: PLoS One. 2017 Mar 8;12(3):e0172594. doi: 10.1371/journal.pone.0172594 (PMC5342202; doi:10.1371/journal.pone.0172594)

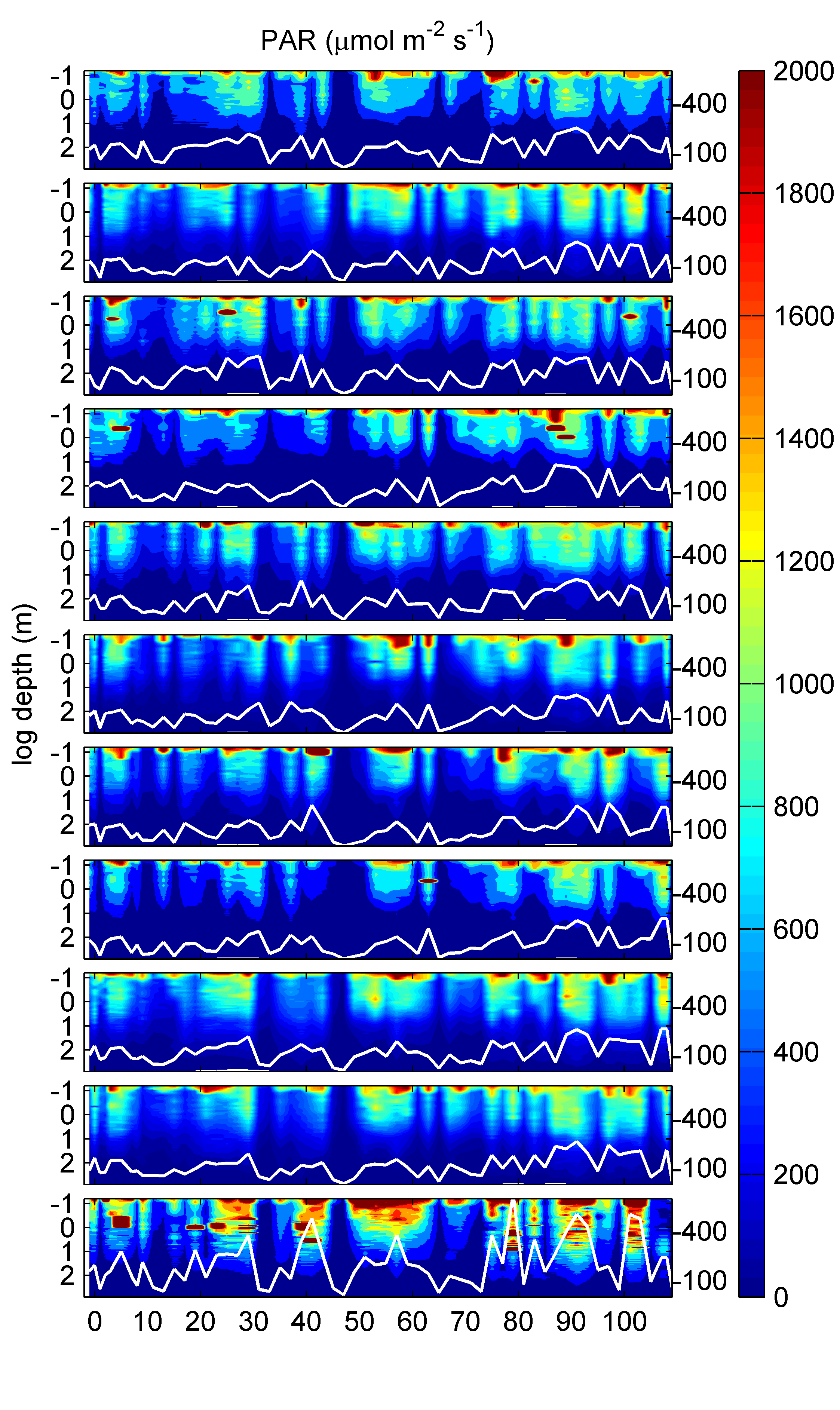

Supplement: S1 Fig — (PNG) [file pone.0172594.s001.png]
